# How 4Ms Age-Friendly Care Led to Improved Compliancy of Older Adult Fall Screenings in Rural Primary Care Clinics

**DOI:** 10.1093/geroni/igab046.3015

**Published:** 2021-12-17

**Authors:** Leah Tobey, Robin McAtee

**Affiliations:** University of Arkansas for Medical Sciences, Little Rock, Arkansas, United States

## Abstract

Past medical history of falls and fear of falling are reliable indicators of future fall risk of an older adult (OA). As one of the HRSA funded Geriatric Workforce Enhancement recipients, the AR Geriatric Education Collaborative (AGEC) worked with a rural federally qualified healthcare clinic system to help incorporate fall screens to satisfy the Mobility factor in the 4Ms age-friendly care framework. After consultation with the practitioners, it was decided to use the Timed-Up-And-Go (TUAG) screen because it is evidence-based and appropriate for OAs. Training on the use of the TUAG was completed next as was the addition of the screen into the EMR. Fall screens in one clinic were only completed 7% before training and 7 months after the training, this rose to almost 100%. In a second clinic, the screens were completed 22% of the time and this was increased to 66% after training. Training on mobility continues to occur on a regular basis as staff turns over and as new priorities arise, but the use of the TUAG as a mobility screen has been a critical component in the process of these rural clinics providing age-friendly care. Next steps with improving fall risks will be the development of flags within the EMR that will force practitioners to complete a full falls plan of care if the OA scored within the moderate or high fall risk categories. The plan will include home safety education and/or evaluation, PT or OT referrals to further support healthy aging for the OA.

